# A Unique Self-Sensing, Self-Actuating AFM Probe at Higher Eigenmodes

**DOI:** 10.3390/s151128764

**Published:** 2015-11-13

**Authors:** Zhichao Wu, Tong Guo, Ran Tao, Leihua Liu, Jinping Chen, Xing Fu, Xiaotang Hu

**Affiliations:** State Key Laboratory of Precision Measuring Technology and Instruments, Tianjin University, Tianjin 300072, China; E-Mails: bertwu@tju.edu.cn (Z.W.); sam1909@163.com (R.T.); liuleihua@tju.edu.cn (L.L.); chenjinping@tju.edu.cn (J.C.); xingfu@tju.edu.cn (X.F.); xthu@tju.edu.cn (X.H.)

**Keywords:** AFM, quartz tuning fork, higher eigenmode, non-contact, finite element analysis

## Abstract

With its unique structure, the Akiyama probe is a type of tuning fork atomic force microscope probe. The long, soft cantilever makes it possible to measure soft samples in tapping mode. In this article, some characteristics of the probe at its second eigenmode are revealed by use of finite element analysis (FEA) and experiments in a standard atmosphere. Although the signal-to-noise ratio in this environment is not good enough, the 2 nm resolution and 0.09 Hz/nm sensitivity prove that the Akiyama probe can be used at its second eigenmode under FM non-contact mode or low amplitude FM tapping mode, which means that it is easy to change the measuring method from normal tapping to small amplitude tapping or non-contact mode with the same probe and equipment.

## 1. Introduction

**Figure 1:**
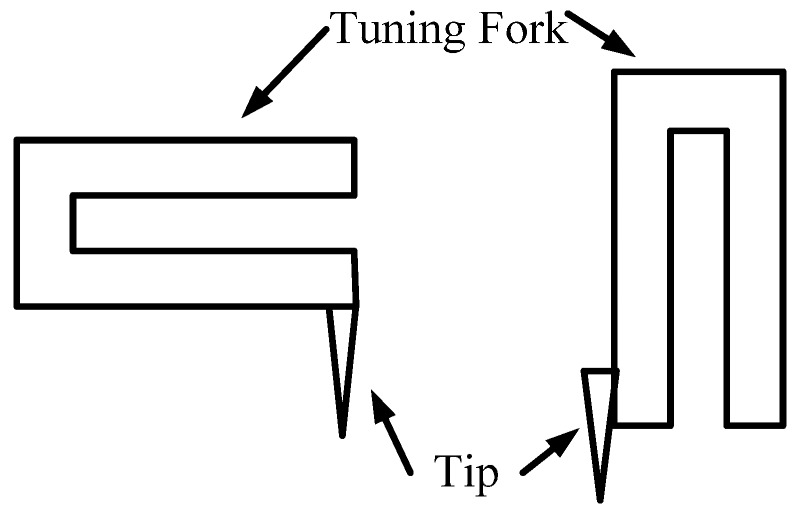
Typical tuning fork probe structures.

Quartz tuning forks are designed for high-precision frequency control and are widely used in clocks, watches, and digital circuit frequency standards. By taking advantage of their extreme stability in frequency, their high quality factor, their self-sensing and self-actuating capabilities, and the ease with which the vibration signal may be obtained with fewer components than the conventional atomic force microscopy (AFM) probes, and so on, they can be used as force sensors in AFM [1,2,3,4]. The tuning fork AFM probes are typically realized in two forms (Figure 1). The tip of the probe could be a carbon nanotube, a fiber, a conventional AFM cantilever, or another type of stylus.

**Figure 2:**
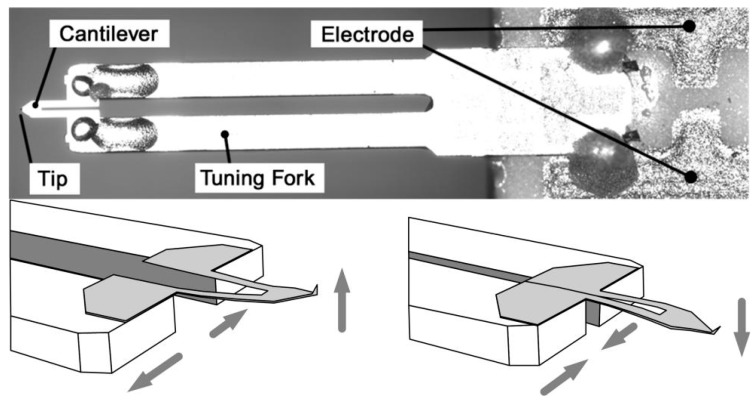
The appearance and motion of an Akiyama probe.

These probes retain their high quality factor and high prong stiffness which makes them a stable source for small vibration amplitudes. On the other hand, the high stiffness is a drawback when measuring soft samples. To couple a soft cantilever to the quartz tuning fork, Bayat *et al.* designed a novel probe [5] which has been commercialized by the Nanosensors Corporation (Neuchatel, Switzerland). As shown in Figure 2, a U-shaped silicon nitride cantilever is combined in a symmetrical arrangement with a quartz tuning fork. A slant probing tip is at the free end of the cantilever. The two legs of the cantilever are fixed to the two prongs of the tuning fork respectively. The parameters of the Akiyama probe are as follows: the resonant frequency is 45–55 kHz; the spring constant is about 5 N/m; the cantilever’s length, width, thickness are respectively 310 μm, 90 μm, 3.7 μm; and the diameter of the tip is less than 15 nm. The tip point is vertical, and lies perpendicular to the lateral plane defined by the tuning fork and cantilever. Under the excitation of the tuning fork, the probe is self-sensing by converting the deflection of the cantilever to a change in charge.

For the long, soft cantilever, this Akiyama probe cannot be used in non-contact AFM at its first eigenmode like the conventional tuning fork probe can [6]. As is well known, a cantilever working at higher eigenmode frequencies can increase the effective stiffness [7,8]. Thus, working at a higher eigenmode can extend the range of application of the Akiyama probe.

## 2. Finite Element Analysis of the Probe

To account for realistic probe geometries and the electric field distribution, finite element analysis (FEA) can be a powerful tool. ANSYS was used in this FEA and the parameters [2] are shown in Table 1.

**Table 1:** FEA parameters.

Parameters	Tuning Fork (Quartz)	Cantilever (SiN)
Elastic constant matrix/GPa	$ANSYS\left[c\right]=\left[\begin{array}{cccccc} c_{11} & c_{12} & c_{13} & 0 & c_{14} & 0\\ & c_{11} & c_{13} & 0 & -c_{14} & 0\\ & & c_{33} & 0 & 0 & 0\\ & & & (c_{11}-c_{12})/2 & 0 & c_{14}\\ & & & & c_{44} & 0\\ & & & & & c_{44} \end{array}\right]$ $\begin{array}{l} c_{11}=86.74,c_{12}=6.99,c_{13}=11.91\\ c_{14}=17.91,c_{33}=107.2,c_{44}=57.94 \end{array}$	None
Piezoelectric constant matrix C/m^2^	$ANSYS\left[e\right]=\left[\begin{array}{ccc} e_{11} & 0 & 0\\ -e_{11} & 0 & 0\\ 0 & 0 & 0\\ 0 & -e_{11} & 0\\ e_{14} & 0 & 0\\ 0 & -e_{14} & 0 \end{array}\right]$ $e_{11}=0.171,e_{14}=-0.0406$	None
Permittivity F/m	4.43, 4.43, 4.63 (*x*-, *y*-, *z*-directions, respectively)	
Young modulus, GPa	None	180
Poisson’s ratio	None	0.28
Density kg/m^3^	2290	2300
Length, μm	2690	310
Width, μm	220	90
Thickness, μm	100	3.7
Finite element	SOLID226	SOLID95

**Figure 3:**
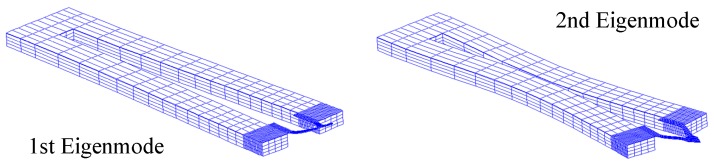
The vibration analysis of the Akiyama probe.

**Figure 4:**
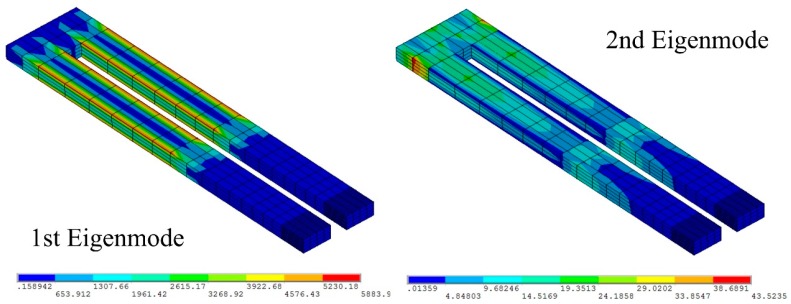
The electric field analysis of the Akiyama probe.

**Figure 5:**
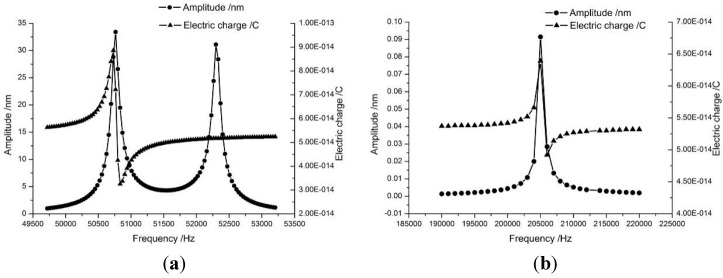
The frequency response curve predicted by FEA. (**a**) The first eigenmode; (**b**) The second eigenmode.

Figure 3 and Figure 4 are the results of the physical and electric analysis at the first and the second eigenmodes respectively. As shown in Figure 5, the vibration amplitude of the second eigenmode is much smaller than that of the first eigenmode, but the electric charge outputs are of the same order of magnitude, which means that the Akiyama probe can obtain detectable low-level outputs.

Conventional small-amplitude AFMs often use short, stiff cantilevers to keep the small vibrations on a low level and ensure that the vibration can be detected. This Akiyama probe, with its long cantilever, may manifest unique behavior while working as a small-amplitude AFM.

## 3. Results and Discussion

**Figure 6:**
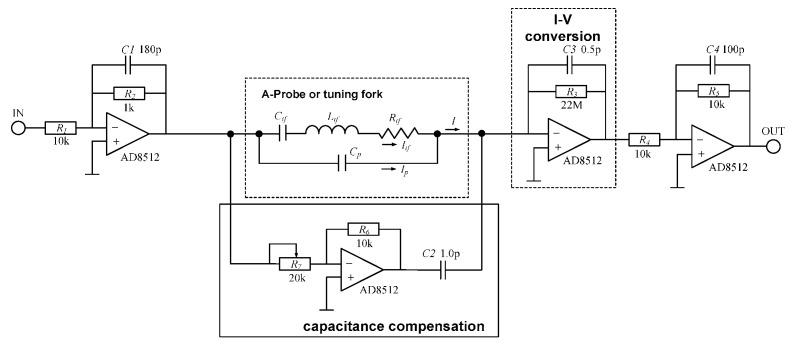
Typical circuit diagram for an Akiyama probe amplification board.

**Figure 7:**
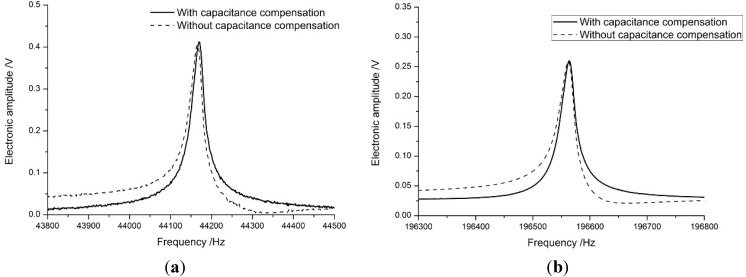
The electrical amplitude-frequency response of an Akiyama probe. (**a**) The first eigenmode; (**b**) The second eigenmode.

The Akiyama probe uses an amplification board based on a typical circuit [2] for I-V conversion and capacitance compensation, as shown in Figure 6 and the influence of the parasitic capacitance is shown in Figure 7.

### 3.1. Frequency Response of the Probe

**Figure 8:**
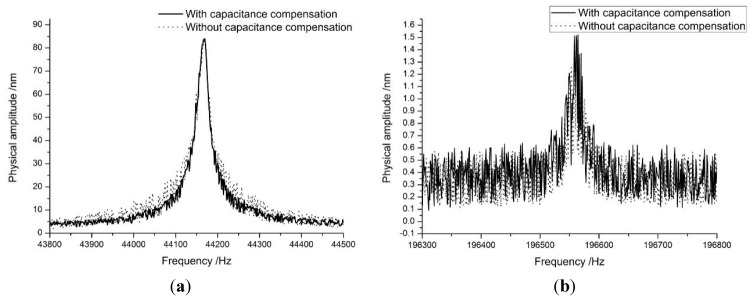
The vibration amplitude-frequency response of an Akiyama probe. (**a**) The first eigenmode; (**b**) The second eigenmode.

The vibration amplitude of the cantilever is measured by laser Doppler vibrometer (LDV). As shown in Figure 8, the amplitude of the first eigenmode is about 85 nm, the second eigenmode’s amplitude is about 1.5 nm which is of the same order of magnitude as the conventional small amplitude AFM. The LDV controller (OFV-3001, Polytec, Waldbronn, Germany) had a velocity decoder resolution of 0.5 μm/s (RMS), a maximum frequency of 250 kHz, and a measuring range of 5 mm/s/V.

**Figure 9:**
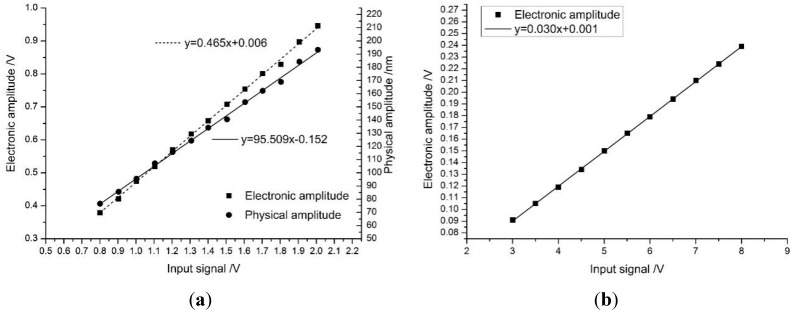
The scale (sensitivity) of the first and second eigenmodes. (**a**) The first eigenmode; (**b**) The second eigenmode.

Differing from the FEA prediction, the electrical output from the second eigenmode was only 10% of that of the first eigenmode, the sensitivity is 0.030 against 0.465 as shown in Figure 9; however, the signal was still easily obtained with the same amplification as that for the first eigenmode, so the amplification board can remain the same for different tests. This meant that the same system could accomplish both normal tapping and small-amplitude tapping modes by changing the excitation signal frequency alone.

### 3.2. The Approach Curve

**Figure 10:**
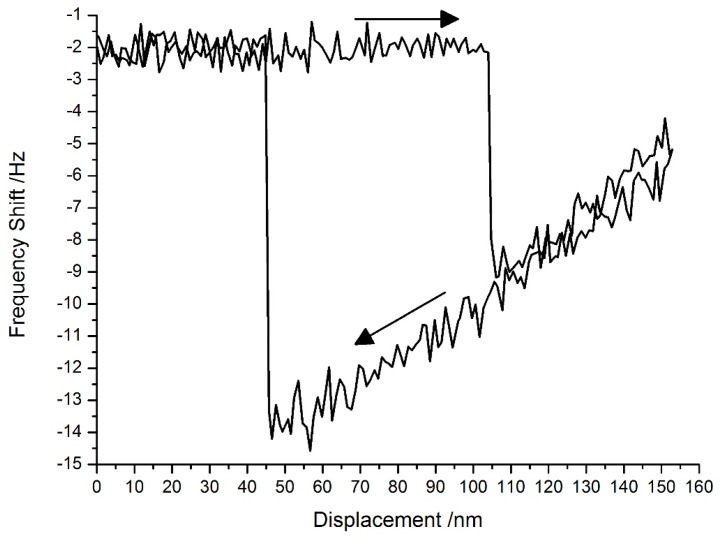
The approach curve for the second eigenmode in FM mode.

The tuning fork probes can work in FM non-contact mode [1]. In the test system, a phase-locked loop (PLL) was used to trace the resonant frequency of the Akiyama probe during its approach to the sample surface. The PLL (HF2PLL, Zurich Instruments, Zurich, Switzerland) had a frequency resolution of 0.8 μHz. The approach curve shown in Figure 10 lay in the attraction region within which non-contact AFM worked.

Compared with the 0.8 Hz/nm sensitivity of the first eigenmode in the repulsion region, the sensitivity of the second eigenmode was about 0.09 Hz/nm; however, under standard atmospheric conditions, the signal-to-noise ratio was not good enough to obtain a higher resolution.

### 3.3. The Resolution of the Probe

**Figure 11:**
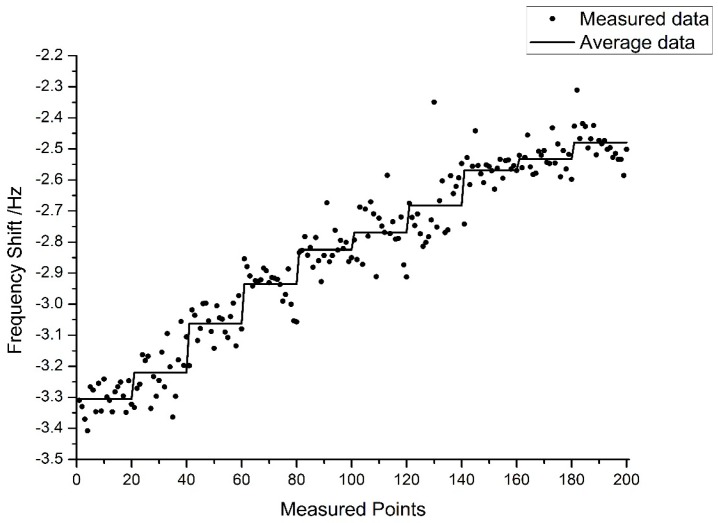
The resolution of an Akiyama probe (2 nm increments).

This test used a piezo-stage to raise the sample in steps of 2 nm after the probe touched the surface with measurements taken at 20 points per step, the measured data and average data of each step are shown in Figure 11.

Although the noise was apparent, the 2 nm steps in the average data plot are visible in Figure 11, which meant that this mode can realize a resolution of 2 nm.

## 4. Conclusions

For a special structure, an Akiyama probe can be used as an FM-mode, non-contact AFM sensor in its second eigenmode, although its cantilever is long and soft. According to the results, the second eigenmode Akiyama probe was similar to the conventional tuning fork AFM probe working under non-contact mode for which the signal-to-noise ratio is not good enough to obtain a better resolution under atmospheric conditions. One advantage of the self-sensing, self-actuating AFM probe based on a tuning fork was the simplicity with which it could be assembled for vacuum AFM to improve the signal-to-noise ratio. Thus, it was appropriate for the measurement of different samples, such as soft or movable samples, and for the use of the Akiyama probe in small-amplitude tapping mode or in non-contact mode, under vacuum, without changing either the probe or the equipment.
